# Uncommon Metastasis of Ovarian Dysgerminoma: A Case Report and Review of the Literature

**DOI:** 10.3390/medicina57060534

**Published:** 2021-05-27

**Authors:** Mihaela Camelia Tîrnovanu, Irina Daniela Florea, Adina Tănase, Bogdan Florin Toma, Elena Cojocaru, Carmen Ungureanu, Ludmila Lozneanu

**Affiliations:** 1 Department of Mother and Child Medicine, “Grigore. T. Popa” University of Medicine and Pharmacy, 700115 Iaşi, Romania; mihaela.tirnovanu@umfiasi.ro (M.C.T.); adinnatanase@gmail.com (A.T.); bogdan-florin-sl-toma@d.umfiasi.ro (B.F.T.); 2 Department of Morphofunctional Sciences I, “Grigore T. Popa” University of Medicine and Pharmacy, 700115 Iaşi, Romania; irina.florea@umfiasi.ro (I.D.F.); ludmila.lozneanu@umfiasi.ro (L.L.)

**Keywords:** dysgerminoma, ovarian germ cell tumors, metastasis, follow-up

## Abstract

Ovarian malignant germ cell tumors (OMGCT) represent less than 10% of all ovarian tumors. Dysgerminoma is the most common malignant primitive germ cell tumor in young women, known for its curability and low propensity to invade and metastasize when diagnosed early. Herein, we report an unusual type of ovarian dysgerminoma (OD) metastasis with a brief review of the literature, lacking similar reported cases. To our knowledge, although there are several case reports of dysgerminoma metastases with variable anatomic location and presentation, vaginal metastasis has not been previously described. The local or systemic relapse together with local and distant metastasis is considered as an independent predictor of poor survival in patients with OD. In light of the absence of mutations status, our patient successfully responded to therapy. Currently, the patient remains in clinical remission. A specific follow-up plan is ongoing knowing that ovarian dysgerminomas tend to recur most often in the first 2–3 years after treatment.

## 1. Introduction

Ovarian dysgerminoma (OD) is a malignant tumor originating from the ovarian primordial germ cells. Unlike other ovarian tumors, dysgerminomas have no precursor lesions. The etiology of OD is not well established. They are defined by the World Health Organization (WHO)as tumors composed of primitive germ cells that do not have a specific pattern of differentiation [1,2]. Dysgerminoma constitute about 0.9–2% of all ovarian malignancies and is about half of malignant ovarian germ cell neoplasms (33–37%) [1,2,3,4,5]. According to a survey performed in the United States comprising 1262 cases of ovarian malignant germ cell tumors (OMGCTs) registered from 1973 to 2002, the age-adjusted incidence of ovarian dysgerminoma per 100,000 women-years was 0.109 [4]. Dysgerminoma is known as ovarian correspondent of testicular seminoma. Germinal cell tumors occur at all ages, but develop especially in children and adolescents with a peak incidence between 10–30 years. Epidemiological data prove that 90% of patients are under 30 years of age, similar to our patient. Tumors diagnosed under the age of five or after menopause are far less common [6]. Still, the literature reports cases of OD in patients aged 7 months to 70 years [7]. Dysgerminoma are responsible for 10% of cancers developed in women younger than 20 years [8].

## 2. Case Presentation

We present a case of a 22-year-old virgin woman admitted to the First Obstetrics-Gynecology Clinic of “Cuza Vodă” Hospital, Iasi, Romania, for subacute pain, palpable abdominal mass with rapid growth and vaginal bleeding for a week. In addition, the patient declared primary amenorrhea. She was born from healthy non-consanguineous parents without significant family history. Informed consent was obtained from the patient involved in the case. The study was approved by the Hospital Ethics Committee (protocol according to the Order 865/22 January 2021).

Physical examination revealed an abdominopelvic solid mass, with irregular borders which extended to approximately 2 cm above the umbilicus. Gynecological examination could not be performed because she had not had sexual intercourse yet. We used for diagnosis the IOTA (International Ovarian Tumor Analysis) criteria based on transabdominal ultrasound (US), computer tomography (CT), and tumor markers [9,10].

**Figure 1:**
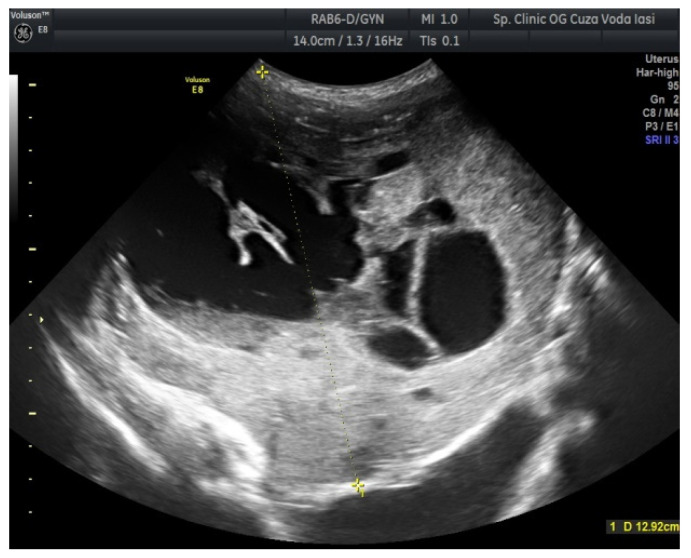
Ovarian ultrasound (US) revealing a large heterogeneous solid mass (right ovary).

**Figure 2:**
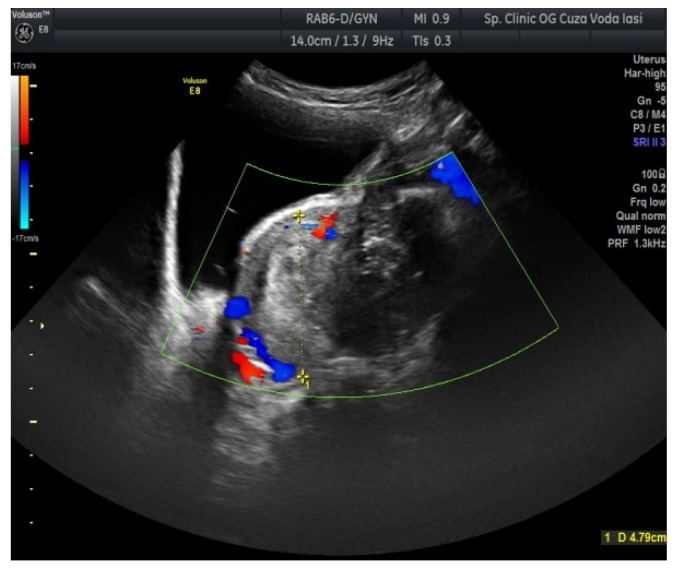
Ovarian US showing a thickened septation with prominent flow signal (right ovary).

**Figure 3:**
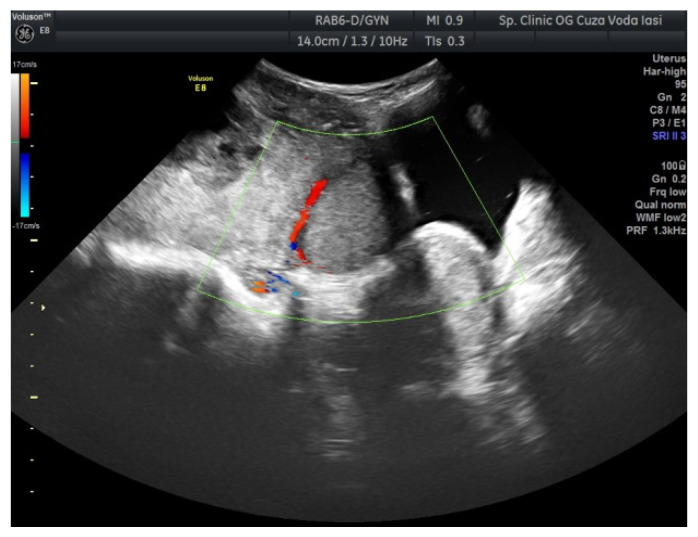
Ovarian color Doppler revealing blood vessels inside the solid part of the tumor (right ovary).

**Figure 4:**
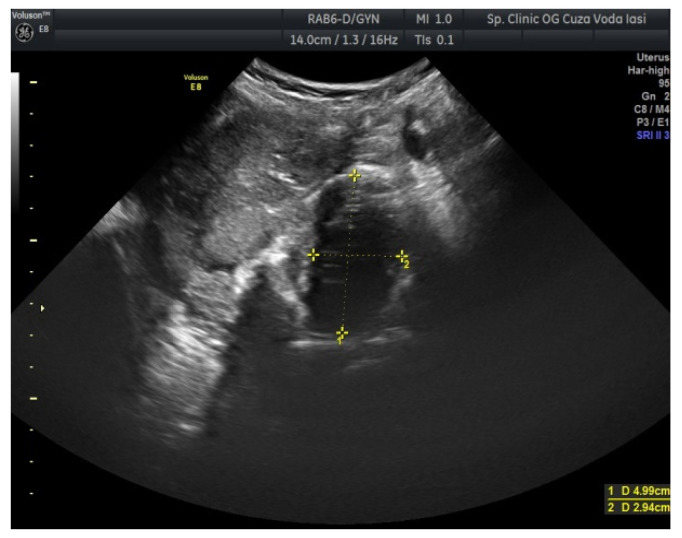
Ovarian US revealing a hypoechoic image (left ovary).

The US showed a large heterogeneous solid mass on the right ovary (Figure 1), about 130 mm with a septate cystic component, showing thickened walls (Figure 2) and prominent flow signal within the septa, suggestive for malignancy. According to the recommendation provided by the IOTA, the first image corresponds to the IOTA forth criteria of malignancy, namely an irregular, multilocular solid tumor with the largest diameter ≥ 100 mm. The US showed no peritoneal effusion. Evaluation with color Doppler revealed blood vessels inside the solid part of the tumor (Figure 3), with a vascular score of 2. The left ovary was slightly increased in dimensions with a hypoechoic image about 50 × 29 mm (Figure 4).

Abdominal CT scan before admission showed: (1) large right ovarian mass (137 × 63 × 54 mm), with solid and liquid components, with vessels and contrast medium uptake; (2) left ovary showing a lesion with polycyclic contour, measuring 56 × 62 × 53 mm with prominent hypodense component, with peripheral solid zones and calcifications in a speckled pattern. Abdominal CT did not display fluid in the peritoneal cavity.

Taking into account the age of the patient, our first hypothesis was ovarian malignant germ cell tumor (OMGCTs). With this in regard, we assessed various serum tumor markers useful for the diagnosis, therapy surveillance, and post-treatment follow-up in OMGCT. It is known that increased values are linked to certain components of OMGCTs. We found an increase in the serum level of lactate dehydrogenase (LDH) of 485 U/L (normal values: 120–246 U/L and human chorionic gonadotropin (hCG) of 56.9 mIU/mL (normal values: <5 mIU/mL). The patient had normal levels of alpha-fetoprotein (AFP) (1.67 IU/mL) and cancer antigen 125 (CA-125) (11.6 U/mL). Two weeks after surgery, the value for lactate dehydrogenase (LDH) decreased to 310 U/L.

Complete blood count (CBC) revealed important anemia with hemoglobin (Hb) values of 8.6 g/dL before surgery and 7.6 g/dL after surgery.

A surgical approach was decided as the first-line treatment. During the median laparotomy, a bilateral ovarian tumor was identified, with no ascites. The maximum dimension of the right ovary tumor was 170 mm with regular surface and the left ovary was very brittle with 60 × 50 mm, adherent to the posterior wall of the uterus. Frozen section revealed malignancy and confirmed bilateral dysgerminoma. Consequently, the patient underwent comprehensive surgical procedures, including total abdominal hysterectomy, bilateral salpingo-oophorectomy with bilateral pelvic lymphadenectomy and peritoneal lavage. A sample of peritoneal lavage fluid was obtained and sent for histological evaluation. No malignant cells were confirmed on cytological findings.

**Figure 5:**
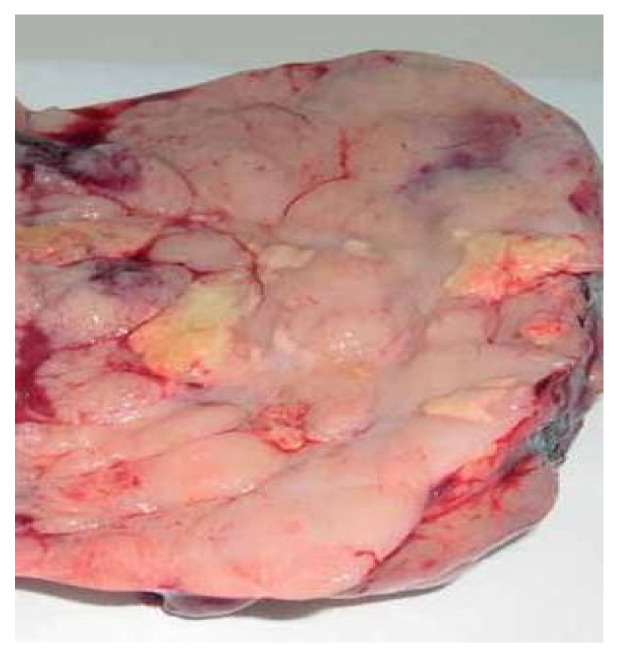
Gross appearance of cut surface of the solid part (right ovary).

Pathological examination described the following gross findings: (i) the right ovary had a solid tumor of 170 × 100 mm with central cystic area of 6 cm, necrosis and hemorrhage; the cut surface of solid part was uniform, pale, gray–pink with a lobe-like pattern (Figure 5); (ii) the left ovary measured 60 × 50 mm, with large area of necrosis (more than 75%) and calcification; (iii) the uterus had serosa involvement; (iv) the right fallopian tube showed tumor invasion at serosa level and necrotic detritus with intraluminal tumor cells, while the left fallopian tube did not present tumor invasion; (v) deposits of necrotic detritus and aggregates of tumor cells (with same tumor features) were identified on cervical adventitia; (vi) infracolic omentectomy—omentum without metastases; (vii) pelvic lymph nodes without metastases (including three lymph nodes at aortic bifurcation).

**Figure 6:**
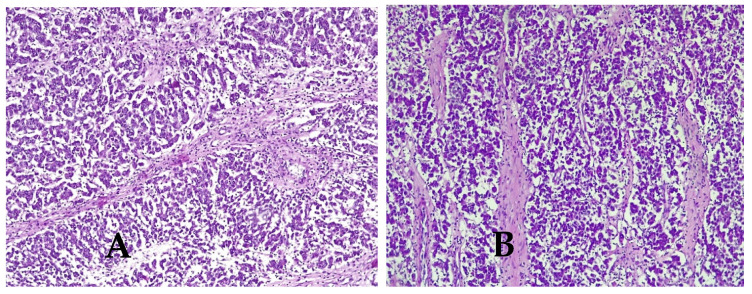

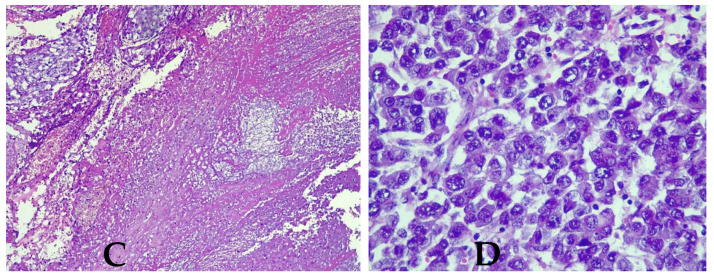
Histologic features of dysgerminoma. (**A**) Nests and nodules of uniform tumor cells separated by. fine connective tissue containing inflammatory cells. Hematoxylin and eosin stain (HE × 10), (**B**) fibrous septa of connective tissue containing lymphocytes (HE × 10), (**C**) large zones of necrosis and hemorrhage (HE × 10), (**D**) tumor cells with nucleus increased in volume with enlarged nucleoli and lymphocytes (HE × 40).

Microscopic aspects were consistent with the diagnosis of dysgerminoma (Figure 6A–D), demonstrating variably sized nests of uniform polygonal cells with abundant granular eosinophilic or clear cytoplasm and distinct cell membranes that resemble primordial germ cells. The cells have a central large round or flattened nucleus that contains one or a few prominent nucleoli. The tumor proliferation has a trabecular, cordonal and pseudoglandular pattern. The tumor architecture presented fibrous septa, with mature lymphocytes. Areas of hemorrhage, coagulative necrosis and cystic changes were seen. High mitotic rate was observed (15 mitoses/10 high power fields). Histopathological examination revealed tumor emboli in adjacent vessels. Final diagnosis was bilateral dysgerminoma, infiltrating the right fallopian tube, serosa of the uterus and cervical adventitia.

The patient was diagnosed according to the latest International Federation of Gynecology and Obstetrics (FIGO) classification with FIGO II A and according to the TNM classification (T—tumor, N—node, M—metastases) with pT2aN0MxL1V1 (p—pathological, T—tumor, N—node, M—metastases, LV—lymphovascular invasion) [1]. No metastases were demonstrated microscopically in any of the retroperitoneal lymph nodes examined.

**Figure 7:**
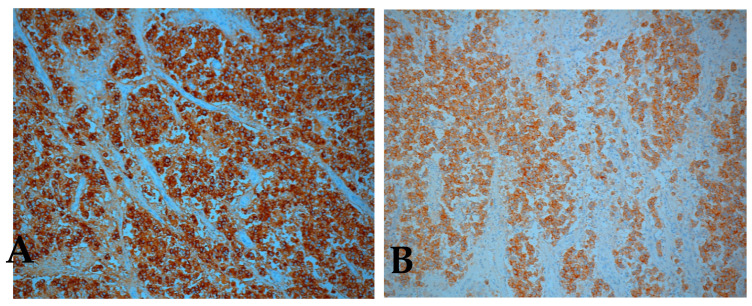
Immunohistochemical staining positive for (**A**) placental alkaline phosphatase (PLAP), (**B**) c-kit / cluster of differentiation 117 (CD117), right ovary (original magnification × 10).

**Figure 8:**
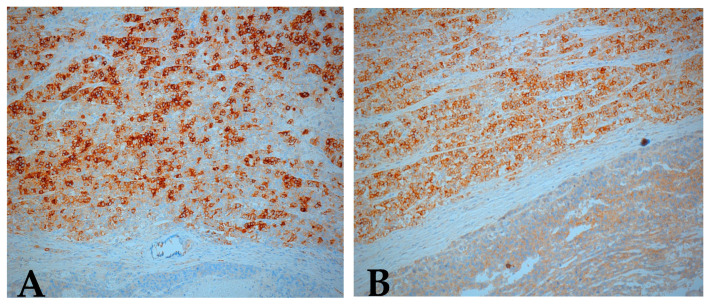
Immunohistochemical staining positive for (**A**) PLAP, (**B**) c-kit (CD117), left ovary (original magnification ×10).

Immunohistochemistry confirmed the bilateral ovarian dysgerminoma. AFP, calretinin, cluster of differentiation 30 (CD30), and inhibin were negative in tumor cells while PLAP (placental alkaline phosphatase) and c-kit/cluster of differentiation 117 (CD117) showed strong positive staining (with membrane enhancement and cytoplasmic expression) in both ovaries (Figure 7A,B). For the left ovary the positivity for PLAP and c-kit was weaker (Figure 8).

**Figure 9:**
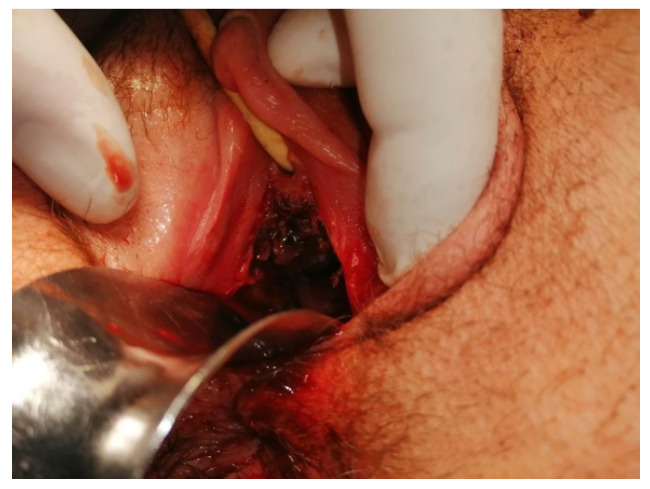
Vaginal metastases of anterior wall.

**Figure 10:**
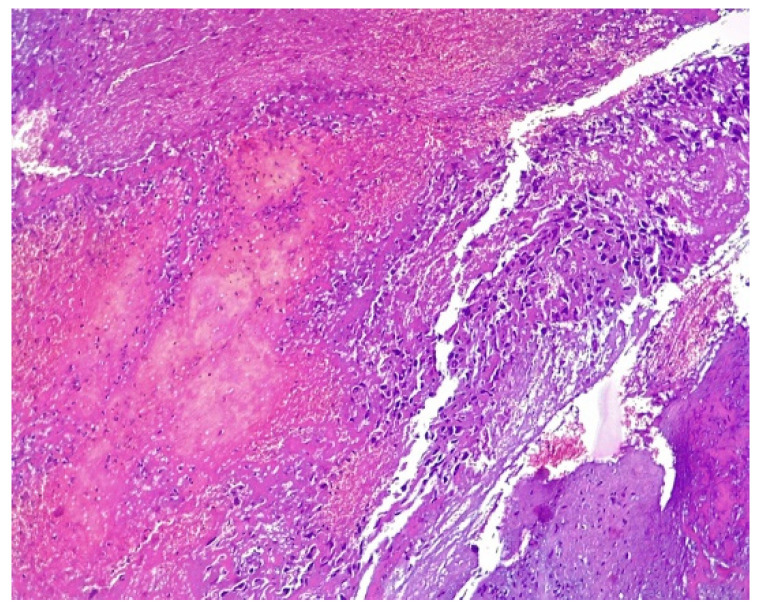
Microscopical aspect of vaginal biopsy with nuclear atypia, pleomorphism, and prominent necrosis, HE × 10.

At 8 days post-surgery, the patient continued to present vaginal bleeding. With general anesthesia, a speculum vaginal examination was performed, identifying a vaginal tumor (Figure 9). Subsequent histological examination described a friable, bloody vaginal metastasis from ovarian dysgerminoma with extensive area of tumor necrosis and hemorrhage (Figure 10). She received blood transfusions and after the final pathological result she was transferred to the Regional Oncological Institute, Iasi, Romania.

**Figure 11:**
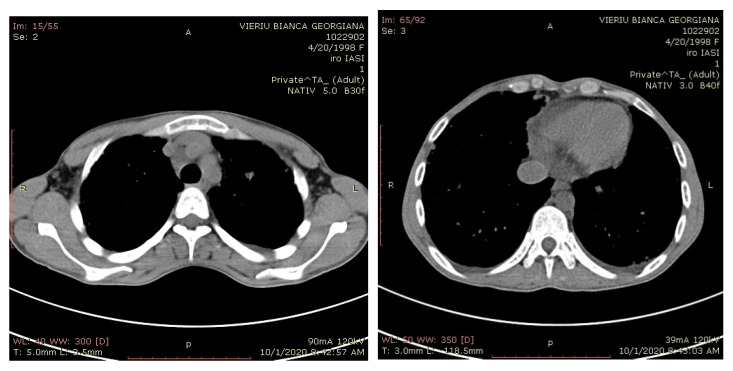
Computer tomography (CT) scans revealed lung metastases.

Thoracic CT after surgery showed multiple pulmonary metastatic micronodular area, disseminated on both lungs in full parenchyma and subpleural (Figure 11). Abdomino-pelvic CT did not identify liver metastasis and retroperitoneal lymph node.

The next step in the management was chemotherapy, including BEP: etoposide, cisplatin and Bleomycin with administration at 3 weeks. The patient tolerated well the induction chemotherapy. The first complete chemotherapy course was started 18 days after surgical intervention. A total of 7 days after the first chemotherapy treatment, only Bleomycin was administered, because the patient presented a severe neutropenia and anemia (Hb = 5.6 g/dL). During this period, the patient was diagnosed with SARS-COV-2 infection.

## 3. Discussion

Germinal cell tumors are usually unilateral, while bilateral neoplasms are rarely reported in about 10–20% [1,11]. The investigations in our case proved the bilaterality of the lesion which led to a more aggressive type of surgery. Usually the cases of bilateral tumors require additional investigations because local expansion with contralateral ovary invasion may be present [7,12].

In the pediatric setting, dysgerminoma has variable clinical manifestations, either asymptomatic (usually presented at stage I of the disease), abdominal distension, abdomino-pelvic mass, ovarian torsion (adnexal torsion), menstrual abnormalities, vaginal bleeding or fever with a median of 2–4 weeks [3,13]. Commonly, the tumoral mass tends to be large at the time of diagnosis and progresses rapidly. Our patient experienced almost all these signs and symptoms. Patients presenting with tumor mass have acute pain due to torsion, rupture of superficial tumor vessels and intra-abdominal hemorrhage. Rare cases of dysgerminoma are associated with ascites and pleurisy. The patient had a minimal bilateral pleural reaction of 9 mm on thoracic CT. Occasionally, dysgerminoma may be diagnosed during pregnancy (20%), with some tumors being fortuitously discovered during cesarean section [14,15].

Regarding imaging features, dysgerminomas are characteristically purely solid with few exceptions. At US, they show heterogeneous echogenicity, smooth lobulated contours and well-defined borders, and they are richly vascularized at color and power Doppler US [16,17]. The US images in our case revealed a heterogeneous solid ovarian mass, with a cystic component and calcifications in a speckled pattern (characteristic of dysgerminoma).

The clinical features and prognosis depend on tumor stage. An overall five-year survival rate is satisfactory exceeding 75% (even 90% in stage I), decreasing to approximately 63% in patients with extended disease beyond the ovaries [18]. The majority of OD are diagnosed at early stage and respond well to fertility-sparing surgery. Localized dysgerminomas are generally accepted to have long-term outcome without recurrence or metastasis. Therefore, dysgerminoma represent a group of potentially curable diseases with excellent prognosis. High responsiveness to chemotherapy may explain the favorable prognosis typically shown in OD. However, their malignant propensity sometimes determines an aggressive course. The local invasion of advanced dysgerminoma causes the destruction of the entire ovarian tissue, the entire genital tract, the lumbar muscles, the iliac vessels, the pubic symphysis, the rectum and the spine [7,19]. Unfortunately, in our case, the involvement of serosa of the uterus, right fallopian tube, vagina along with the transition from FIGO IIA stage to FIGO IV by the presence of distant metastases proved an advanced disease. Moreover, our patient’s chemotherapy treatment was administered discontinuously and in lower doses due to COVID–19 infection during hospitalization. In addition, this unusual presentation with vaginal and lung metastasis is considered a significant predictor of unfavorable prognosis.

Other factors that can be associated with reserved prognosis include: tumor stage, histological type, value of tumor markers and the presence of residual tumor. In cases where the tumor is well defined, surgical removal is not generally followed by local recurrences or metastases. Still, tumor recurrences occur in approximately 10–20% of patients in the vast majority of cases in the stage I and first two years of initial presentation [1,20,21]. The recurrence rate increases significantly after the age of 45 [12]. At the present time, there is no evidence of recurrent disease in our patient.

In the advanced stage, dysgerminoma infiltrates the neighboring tissues by direct extension. It also spreads into the regional or distant lymph nodes and at the level of the pelvic and abdominal peritoneum [22,23]. Dissemination by hematogenous invasion is responsible for distant, uncommon metastasis [22]. Our case did not have lymph node metastasis.

The most common metastases of dysgerminoma are the peritoneal cavity, omentum (86%), pelvis and abdomen [11,23], and retroperitoneal lymph node [11,23,24]. Furthermore, extra-abdominal lymph nodes such as para-aortic, [23,25,26], supraclavicular [11] and cervical lymph nodes [26,27,28] may be involved. Distant hematogenous metastases develop in the bones [29,30,31], lungs (15%) [24], liver [29,31], kidneys [29], adrenal glands [31], mammary glands [32], cranium, brain, dura mater and intra-axial [24,33,34], pancreas [29], heart and skin [32]. There are also a number of other uncommon distant sites that have been reported: eye, placenta, central airways (bronchus-trachea), bladder (17%), pleura (33%), spleen (20%), bowel (50%) [22,35], and neurologic involvement such as carcinomatous meningitis [24,33,34]. Our literature search did not reveal vaginal metastasis from OD, as reported in our patient, explaining our interest for the case. Vaginal metastasis was not diagnosed at the initial presentation because the patient was a virgin. Initially, we questioned whether vagina involvement is the result of metastatic disease by lympho-vascular spread or local extension from adjacent OD. It was certain that the histological characteristics of the vaginal metastasis were similar to the primary ovarian tumor. Given the presence of vascular tumor emboli, we assumed that the vaginal infiltration represents hematogenous spread of the OD. In addition, we discovered a distant hematogenous lung metastasis.

The majority of reported vaginal metastasis are derived from cervical (51%), endometrial (13.3%), ovarian cancer [36,37,38], kidney (1.3%) [39], colon [40], breast, pancreas [41] or bladder cancer. We would like to mention that some rare metastases were selected from articles that did not mention with certainty the type of ovarian tumor.

A study performed in 2020 by Maekawa et al. revealed a cervical lymph node metastasis from OD on fine needle aspiration cytology [28]. Kumar et al. reported that the prevalence of lymph node metastasis predicts recurrences and metastasis in patients with OD [42]

Epidemiological data showed that OD can be associated with some factors, such as (i) dysontogenic gonads; (ii) paraneoplastic hypercalcemia or (iii) endocrine disorders. Recent evidence suggests that OD is the most common malignant tumor of the gonads in patients with dysgenetic gonads. According to the WHO Classification of Tumors 2020, OD usually occurs as a part of gonadal dysgenesis [1]. Tumors can be discovered by chance during investigations to determine causes of primary amenorrhea in women with (i) Swyer syndrome (46 XY karyotype), known as pure gonadal degeneration, (ii) mixed gonadal dysgenesis (45X/46XX) or (iii) partial gonadal dysgenesis (46XX) [43]. For this reason, dysgerminoma has a particularly high frequency of gonadal neoplasia, dictating early prophylactic removal of these dysgenetic gonads [1,7]. Previous findings [44] showed that patients with Swyer syndrome have a risk of dysgerminoma development, with bilateral gonadectomy being recommended in such cases. Howitt et al. reported that the incidence of dysgeminoma in people with abnormal genital development or chromosomal abnormalities is about 5–10% [45]. Even though our patient presented with primary amenorrhea, we had no data regarding genetic testing. Within this framework, we recommend a mutational screening as useful for other family members. However, diagnostic molecular pathology has no clinical relevance in OD as stated by WHO Classification of Tumors 2020 [1].

Previous data reported associations of dysgerminoma with paraneoplastic hypercalcemia [1,11,21,46,47]. Furthermore, a recently published study sustains that hypercalcemia is associated with elevated parathyroid hormone, parathyroid hormone-related protein (PTH-rP), 1,25 dihydroxyvitamin D [47] and hemophagocytic lymphohistiocytosis. We had no information regarding PTH level in our patient.

Dysgerminoma is not a hormone-secreting tumor and is not usually accompanied by endocrine disorders. Still, in some cases it is accompanied by endocrinal manifestations such as early puberty, hirsutism or signs of virility. In connection with these issues, our patient presented primary amenorrhea that could be induced by increased testosterone, but we did not investigate it. The patient reported the lack of menstruation up to 22 of age. However, she had an endocrinological consult at the age of 15, but she was not directed to a pelvic ultrasound or gynecological consult.

Some patients with dysgerminoma (5%) have slightly elevated human chorionic gonadotropin (hCG) levels secreted by syncytiotrophoblastic giant cells [1,7,13,48]. Estrogenic hormonal manifestations such as sexual pseudo precocity, menstrual disorders and vaginal bleeding or pregnancy symptoms are usually caused by elevated serum β-hCG levels [49]. Although the patient in this case did not reveal pregnancy symptoms, she presented elevated β-hCG level. It was proved that elevated hCG levels or elevated α-fetoprotein (αFP) concentrations suggest the association of other germ cell tumors such as choriocarcinoma, yolk sac tumor or immature teratoma. In the present case, the association was not identified. In addition, serum LDH is often elevated and can also serve as a tumor marker. A correlation was found between plasma level and tumor stage, similar to our patient in which LDH levels decreased almost to a normal value two weeks after surgery. Moreover, these markers are useful in monitoring therapy, but also in identifying recurrences [45]. For this reason, elevated hCG levels may raise the suspicion of ovarian cancer, but a negative result does not rule out the diagnosis. In the case of ovarian dysembryoplastic tumors, the increase in serum of β-hCG has a 86% sensitivity. Song et al., 2007, reported the case of a 6-year-old girl with precocious puberty and high levels of β-hCG, αFP and estradiol who was diagnosed with OD with syncytiotrophoblastic giant cells (SyTGC). After resection of the tumor, the levels of β-hCG and αFP markers became normal and precocious puberty disappeared. Our patient did not present with SyTGC. Song et al. recommend resection of the tumor because of the high risk of malignant transformation of SyTGC [50].

The morphological landscape and structural heterogeneity of these tumors is the consequence of germ cells’ ability to differentiate in divergent ways at different stages of development [49]. Histologically, dysgerminoma is divided into a pure form, having a good prognosis, and a mixed-type with a variable admixture of germinal elements (15%) (teratoma, embryonic carcinoma or yolk sac tumor) [1,4]. Even though our patient had a pure dysgerminoma, the vascular invasion dictates the prognosis.

## 4. Conclusions

To the best of our knowledge, our patient is the first reported case with vaginal metastasis of ovarian OD in the clinical scenario. The patient had genital bleeding because of vaginal tumor implants. OD should be included in the differential diagnosis for a young female who presents non-acute lower quadrant pain, palpable pelvic mass and elevated β-hCG and LDH. If the patient had been investigated for amenorrhea, the tumors could have been identified in an earlier stage. Quantification of the tumor markers β-hCG and LDH at diagnosis allows for monitoring of the tumor recurrence after treatment. Distinct histopathological features and immunohistochemistry were helpful for an accurate diagnosis for bilaterality in our case. The majority of dysgerminomas are diagnosed in early stage, unlike our case who presented in an advanced stage with lung metastasis favoring a poor outcome. Prognosis can be good with a good five-year survival after appropriate chemotherapy. The risk for recurrence increases in the first 2–3 years after treatment, so a close follow-up in our case is required.

## Data Availability

The data presented in this study are available on request from the corresponding author. The data are not publicly available due to privacy.
